# Apolipoprotein A-I Is a Potential Mediator of Remote Ischemic Preconditioning

**DOI:** 10.1371/journal.pone.0077211

**Published:** 2013-10-14

**Authors:** Pierre Hibert, Delphine Prunier-Mirebeau, Olivia Beseme, Maggy Chwastyniak, Sophie Tamareille, Delphine Lamon, Alain Furber, Florence Pinet, Fabrice Prunier

**Affiliations:** 1 L’UNAM Université, Angers, France; 2 Laboratoire Cardioprotection, Remodelage et Thrombose, Université d’Angers, Angers, France; 3 INSERM U771, CNRS UMR 6214, Département de Biochimie et Génétique, Université d’Angers, CHU Angers, Angers, France; 4 INSERM, U744, Lille, France; 5 Institut Pasteur de Lille, Lille, France; 6 Université Lille Nord de France, IFR142, Lille, France; 7 Service de Cardiologie, CHU Angers, Angers, France; 8 Centre Hospitalier Régional et Universitaire, Lille, France; I2MC INSERM UMR U1048, France

## Abstract

**Background:**

Remote ischemic preconditioning (RIPC) has emerged as an attractive strategy in clinical settings. Despite convincing evidence of the critical role played by circulating humoral mediators, their actual identities remain unknown. In this study, we aimed to identify RIPC-induced humoral mediators using a proteomic approach.

**Methods:**

and Results Rats were exposed to 10-min limb ischemia followed by 5- (RIPC 5′) or 10-min (RIPC 10′) reperfusion prior to blood sampling. The control group only underwent blood sampling. Plasma samples were analyzed using surface-enhanced laser desorption and ionization - time of flight - mass spectrometry (SELDI-TOF-MS). Three protein peaks were selected for their significant increase in RIPC 10′. They were identified and confirmed as apolipoprotein A-I (ApoA-I). Additional rats were exposed to myocardial ischemia-reperfusion (I/R) and assigned to one of the following groups RIPC+myocardial infarction (MI) (10-min limb ischemia followed by 10-min reperfusion initiated 20 minutes prior to myocardial I/R), ApoA-I+MI (10 mg/kg ApoA-I injection 10 minutes before myocardial I/R), and MI (no further intervention). In comparison with untreated MI rats, RIPC reduced infarct size (52.2±3.7% in RIPC+MI *vs*. 64.9±2.6% in MI; *p*<0.05). Similarly, ApoA-I injection decreased infarct size (50.9±3.8%; *p*<0.05 *vs*. MI).

**Conclusions:**

RIPC was associated with a plasmatic increase in ApoA-I. Furthermore, ApoA-I injection before myocardial I/R recapitulated the cardioprotection offered by RIPC in rats. This data suggests that ApoA-I may be a protective blood-borne factor involved in the RIPC mechanism.

## Introduction

Local ischemic preconditioning, which consists of transient non-lethal episodes of myocardial ischemia before a prolonged ischemia-reperfusion (I/R) cardiac injury, is well recognized as one of the most potent innate cardioprotective mechanisms. However, it necessitates direct application of invasive procedures to the myocardium in order to attain cardioprotection, which could be harmful in clinical settings. An alternative strategy is to apply the cardioprotective stimulus to an organ or tissue far from the heart, an approach entitled remote ischemic preconditioning (RIPC) [1], [2]. Short periods of I/R of various remote tissues, including the intestine [3], kidney [3], [4], and skeletal muscle [5], were reported to protect the myocardium and other tissues from I/R injury [6]. With no need for invasive procedures, RIPC using transient limb ischemia as a stimulus has emerged as an attractive strategy in clinical settings [2], [7]. This strategy was shown to attenuate myocardial injury in patients undergoing corrective cardiac surgery for congenital heart disease [8], coronary bypass surgery [9], elective surgery for abdominal aortic aneurysm [10], and elective percutaneous coronary interventions (PCI) [11]. Recently, intermittent arm ischemia with four cycles of 5-min inflation and 5-min deflation of a blood pressure cuff, in the ambulance during transfer to primary PCI, has been shown to significantly increase myocardial salvage [12]. The protective mechanisms underlying remote ischemic conditioning are still unclear. However, there is convincing evidence for the critical role played by humoral mediators transported through the circulation [13]–[16]. Yet, the actual identity of the humoral mediators remains undetermined.

Proteomic analysis provides an expanded approach to the study of biomarkers selected to a non *a priori* basis. Surface-enhanced laser desorption and ionization - time of flight - mass spectrometry (SELDI-TOF-MS), which is a combination of both chromatography and mass spectrometry (MS), is a sensitive method for accessing proteolytic fragments from major plasma proteins [17]. Therefore, this study was designed to identify potential RIPC-induced humoral mediators via SELDI-TOF-MS technology.

## Materials and Methods

### Ethics Statement

All experiments were performed in accordance with the *Guide for the Care and Use of Laboratory Animals* published by the US National Institute of Health [NIH publication 85 (23), revised in 1996]. Protocols have been approved by our regional ethic committee: Comité Régional d’Ethique pour l’Expérimentation Animale-Pays de la Loire (N°CEEA.2012.50). All surgery was performed under sodium pentobarbital anesthesia, and all efforts were made to minimize suffering.

### Animal Studies

Two animal studies were conducted. Study 1 was performed in order to identify RIPC-induced circulating factors by proteomic analysis. Study 2 was carried out in order to verify the potential cardioprotective effect of the circulating factor identified, notably apolipoprotein A-I (ApoA-I), in a myocardial I/R model.

For Study 1, 8- to 10-week-old male Wistar rats were anesthetized with 60 mg/kg of intraperitoneal sodium pentobarbital (Céva santé Animal, France) and orotracheally intubated with a 16-gauge tube. Animals were ventilated using a small animal ventilator at a rate of 50–60 breaths per minute (SAR-830 A/P, CWE). The body core temperature was continuously monitored during the procedure and maintained at 37±1°C using a homeothermic blanket, connected to a temperature control unit (HB101/2 RS, Bioseb, France).

Rats were randomly assigned to one of three groups: Control (which endured no further intervention, but blood sampling only, n = 10); RIPC 5′ (blood sampling 5 minutes after the end of limb ischemia, n = 10); RIPC 10′ (blood sampling 10 minutes following the end of limb ischemia, n = 10). RIPC was achieved using a vascular clamp placed on the upper right femoral artery in order to occlude arterial blood flow for 10 minutes, followed by reperfusion. Limb ischemia was confirmed by a change in skin color and a decrease in subcutaneous limb temperature. Following limb reperfusion, the skin color turned back to a pink color, while the under-skin temperature reached the baseline temperature. Blood samples were collected from the inferior vena cava 5 or 10 minutes after limb reperfusion using glass tubes that contained the anticoagulant ethyldiamine tetraacetic acid (EDTA). Plasma samples were immediately processed, divided into aliquots of 500 µL, and stored at −80°C. These samples underwent no more than two freeze/thaw cycles prior to analysis.

For Study 2, 8- to 10-week-old male Wistars rats were used. All animals were anesthetized via intraperitoneal injection of sodium pentobarbital (60 mg/kg) (Céva santé Animal) and endotracheally intubated with a 16-gauge tube. Animals were ventilated using a small animal ventilator (SAR-830 A/P, CWE). Body core temperature was continuously monitored throughout the surgical procedure and maintained at 36–38°C using a homeothermic blanket, connected to a temperature control unit (HB101/2 RS, Bioseb, France). The chest was opened via median sternotomy. The pericardium was removed, the heart was exposed, and a 7.0 monofilament suture (Premio 7.0, Peters Surgical) was placed around the proximal portion of the left anterior descending coronary artery (LAD) and passed through a short piece of tubing (PE50) in order to create a reversible snare. Following cardiac stabilization, coronary occlusion was initiated by clamping the snare onto the epicardial surface directly above the coronary artery. Ischemia was confirmed by epicardial cyanosis below the suture and dyskinesis of the ischemic region. Following 40 minutes of occlusion, reperfusion was achieved by loosening the snare and confirmed by a marked hyperemic response on reperfusion. Three groups were constituted: MI (myocardial I/R alone, n = 11); RIPC+MI (10-min limb ischemia followed by 10-min reperfusion initiated 20 minutes before coronary artery occlusion, n = 13); ApoA-I+MI (ApoA-I injection 10 minutes before coronary artery occlusion, n = 10). RIPC was performed as described in Study 1. ApoA-I (Academy Bio-Medical Company, Inc. Houston, TX, USA) was injected intravenously as a bolus of 10 mg/kg. The dose was chosen to assess ApoA-I afforded cardioprotection in accordance with the literature that describes protective effects of ApoA-I in a range from 5 mg/kg to 25 mg/kg [18]–[20]. Infarct size was measured at the end of the 2-hour reperfusion period.

### Area at Risk and Infarct Size Determination

The heart was removed and the LAD reoccluded using the monofilament suture kept in place. The heart was then retrogradely perfused with Evans blue (1%) in order to delineate the area at risk (AAR). The heart was then cut into five to six slices from the apex to the base followed by incubation at 37°C in a 1% solution of phosphate-buffered 2,3,5-triphenyltetrazolium chloride (TTC, Sigma-Aldrich, St Louis, MO, USA) so as to delineate the infarcted myocardium. Slices were then fixed in 10% formalin, and the infarct size was quantified by computerized planimetry using image J software (National Institutes of Health, Bethesda, MD). The area of necrosis was expressed as a percentage of AAR (INF%AAR), and AAR as a percentage of the total left ventricular area (AAR%LV).

### Seldi-TOF-MS Profiling

Samples (200 µL) were pretreated with the ProteoMiner® protein enrichment kit, based on a combinatorial peptide ligand library (CPLL) (Bio-Rad Laboratories, Hercules, CA), as previously described [21]. Elution samples (20 µL repeated three times) were pooled and stored at −20°C.

Collected samples were profiled using SELDI-TOF-MS analysis. Native and CPLL treated forms of all samples were analyzed using CM10 (weak cation exchanger) and H50 (reverse phase) ProteinChip® Arrays (Bio-Rad Laboratories). Each of these samples was tested in duplicate and randomly distributed on arrays, as previously described [21]. All data was processed using ProteinChip® Data Manager software. Peaks were detected automatically. Groups of peaks with similar mass across the spectra were assembled into clusters, according to two-step parameter settings, as previously described [21]. The *m/z* range was set between 3,000 and 30,000 for low-mass proteins, and between 20,000 and 150,000 for high-mass proteins.

Proteins corresponding to the peaks found at *m/z* 27583–27791 and 28284 were isolated and purified by liquid-phase isoelectric focusing (IEF) via the MicroRotofor cell (Bio-Rad Laboratories), as previously described [22]. A 30 µL CPLL-treated plasma sample was diluted with an IEF buffer solution (7 mol/L urea, 2 mol/L thiourea, CHAPS 4% [w/v], and 0.24% Triton X100), glycerol (5% v/v), and ampholytes (pH range 4–6, 1.6% v/v). Focusing was performed at room temperature, under a constant power of 1W. At the end of IEF, protein fractions from each compartment (200 µL) were harvested quickly in order to avoid the diffusion of separated proteins, and a 2-D Clean-Up kit (GE Healthcare) was used in order to precipitate proteins. Precipitation was performed to selectively discard contaminants from the proteins. Finally, the pellet was suspended in 20 µL of de-ionized water and stored at −20°C. The detection of peaks (5 µL) was performed on ProteinChip® NP20 array.

Fractions containing the purified peaks were separated on a 15% SDS-PAGE gel (Bio-Rad Laboratories), stained with Coomassie Brilliant Blue, as described by Neuhoff *et al.* [23].

### Protein Identification by Mass Spectrometry

Proteins were then identified by an in-gel digestion procedure, as previously described [24]. Briefly, the band of interest was excised, and the gel plugs were washed with ultrapure water until the stain was completely removed. Next, gel excisions were rinsed several times and rehydrated with a solution containing 0.025% ProteasMAX™ Surfactant, Trypsin Enhancer (Promega) in 50 mM ammonium bicarbonate, and 3 µL of 40 µg/mL Trypsin Gold (Promega) in 50 mmol/L acetic acid. Following overnight digestion at 37°C, peptide extraction was carried out in a two-step procedure in accordance with the manufacturer’s protocol for ProteasMAX™ Surfactant, Trypsin Enhancer. For acquisition of the mass spectra of the extracted and desalted peptides, 0.5 µL of the peptide solution was mixed with 0.5 µL of matrix solution (5 mg/mL of α-cyano-4-hydroxycinnamic acid dissolved in 0.1% TFA/50% ACN) on the matrix assisted laser desorption and ionization – time of flight – mass spectroscopy (MALDI-TOF-MS) target. External calibration was performed with a peptide mixture resultant from the tryptic digest of BSA (0.5 µg/mL). MALDI-TOF-MS was then performed with a Voyager DE STR mass spectrometer (PerSeptive Biosystems, Framingham, MA, USA) equipped with a 337.1 nm nitrogen laser and a delayed extraction facility (125 msec). All spectra were acquired in a positive ion reflector mode at the voltage of 20 kV, with grid-voltage of 61%. Typically, 300 laser shots were recorded per sample. Following this, the mass spectra were calibrated prior to protein identification by peptide mass fingerprinting, conducted by running the MASCOT web searcher (http://www.matrixscience.com/, Matrix Science, UK) against the Swissprot 57-15 (515203 sequences; 181334896 residues) with the following parameters: fixed modifications: carbamidomethyl (C) and variable modifications: oxidation (M); peptide mass tolerance: ±50 ppm; peptide charge state: 1+; max missed cleavages: 1; taxonomy: rattus.

Identification was validated by immunodepletion using a specific rabbit anti-apolipoprotein A-I antibody (Bioss Antibody: bs-0849R). A sample of 10 µg of antibody was incubated with 5 µL of native plasma in 500 µL of IP buffer (Triton X-100 1%, NaCl 150 mmol/L, EDTA 1 mmol/L, EGTA 1 mmol/L, sodium vanadate 0.1 mmol/L, and NP40 0.5% in Tris-HCl 10 mmol/L) at 4°C, as previously described [25]. Subsequent to overnight incubation, 50 µL of protein A Sepharose 4 Fast Flow beads (GE Healthcare) were added, and the preparation was rotated end-to-end for 4 hours at 4°C. Antibodies were removed from the plasma using treated protein A-sepharose beads and saved. Immunodepleted and undepleted plasma were compared by SELDI analysis on ProteinChip® H50 array; a loss of the peak of interest was visualized when compared to untreated plasma.

### Validation by Quantification of Plasma Apolipoprotein A-I by ELISA

Concentrations of ApoA-I were determined by Enzyme-linked Immunosorbent Assay (ELISA) kit for rat apolipoprotein A-I (USCN, Life Science Inc., Wuhan, China) [26], [27], in the 30 plasma samples obtained from the rats used in the proteomic study. The ApoA-I ELISA test has a sensitivity of 6.2 ng/mL and is reported to exhibit no cross-reactivity with other analogues.

### Statistical Analysis

All values were expressed as mean ±SEM. Statistical analyses were performed using SPSS 17.0 (SPSS Inc, Chicago, IL, USA). Due to its nonlinear distribution, we elected to apply a non-parametric test for independent samples on ApoA-I quantification by ELISA. We performed the Kruskal-Wallis test followed by a Mann-Whitney test. After testing their linear distribution, differences between infarct sizes were evaluated using one-way ANOVA, followed by the *post-hoc* Bonferroni test. Pearson’s chi-squared test was used to compare mortality rates between groups. Clusters of all spectra obtained using SELDI-TOF-MS were subjected to univariate analysis, with a non-parametric test to calculate the *p* value of each cluster, and a Mann-Whitney test to compare groups in pairs. A *p* value <0.05 was considered statistically significant.

## Results

### Identification of Apolipoprotein A-I as an RIPC-induced Circulating Factor in Rats

Profiling of plasma samples (CPLL-treated or in native form) from Control, RIPC 5′, and RIPC 10′ groups was performed in duplicate using SELDI-TOF-MS technology on ProteinChip® CM10 or H50 arrays. From a total of 207 peaks detected for low-mass proteins (89 for CM10, 118 for H50) and 164 peaks for high-mass proteins (74 for CM10, 90 for H50), 30 SELDI peaks displayed significant modulation relative to their RIPC groups, with 15 being increased and 15 decreased.

**Figure 1:**
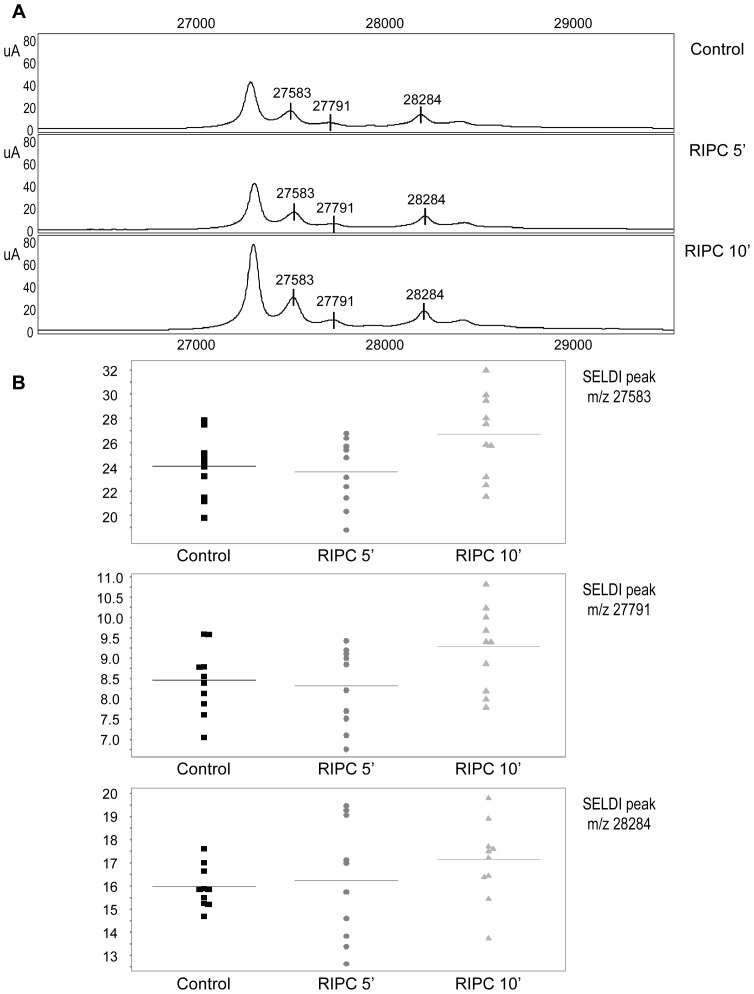
Illustration of protein spectra and m/z means of cases and controls. (A) Representative SELDI-TOF-MS protein spectra of plasma sample from one Control, one RIPC (remote ischemic preconditioning) 5′, and one RIPC 10′ rat. Results are presented as intensities of SELDI-TOF reading (arbitrary units). The 27583, 27791, and 28284 *m/z* peaks were found to be differentially expressed on the CM10 array as calculated by the Mann-Whitney test. (B) Scattergram showing the significant differences in intensity of 27583, 27791, and 28284 *m/z* peaks in plasma samples derived from Control, RIPC 5′, and RIPC 10′ rats. The continuous line represents the mean, and dots represent each individual rat (n = 10 in each group). Detailed *p-*value data for comparison between the three groups is indicated in Table 1.

We focused on three peaks, exhibiting 27583, 27791, and 28284 *m/z,* with significantly different mean levels between the RIPC 5′ and RIPC 10′ rats (**Table 1**). **Figure 1A** presents a detailed representative spectrum for one rat from each group, Control, RIPC 5′, and RIPC 10′. **Figure 1B** demonstrates the individual intensity for each peak in the three groups. Interestingly, we observed an increased intensity for the three peaks at 10-min reperfusion compared to 5-min reperfusion, in favor of protein release.

**Table 1:** SELDI profiles of plasma from Control, RIPC 5′ and RIPC 10′ rats.

Peaks	Control	RIPC 5′	RIPC 10′
*m/z* (Da)	(n = 10)	(n = 10)	(n = 10)
27583	24.03±2.63	23.56±2.73	26.71±3.45*
27791	8.45±0.81	8.31±0.96	9.28±1.02*
28284	15.98±0.90	16.24±2.56	17.14±1.72†

Data are mean±SEM. Da: Daltons.

* *p*<0.05 *vs.* RIPC 5′,

† *p*<0.05 *vs.* Control.

RIPC indicates remote ischemic preconditioning.

**Figure 2:**
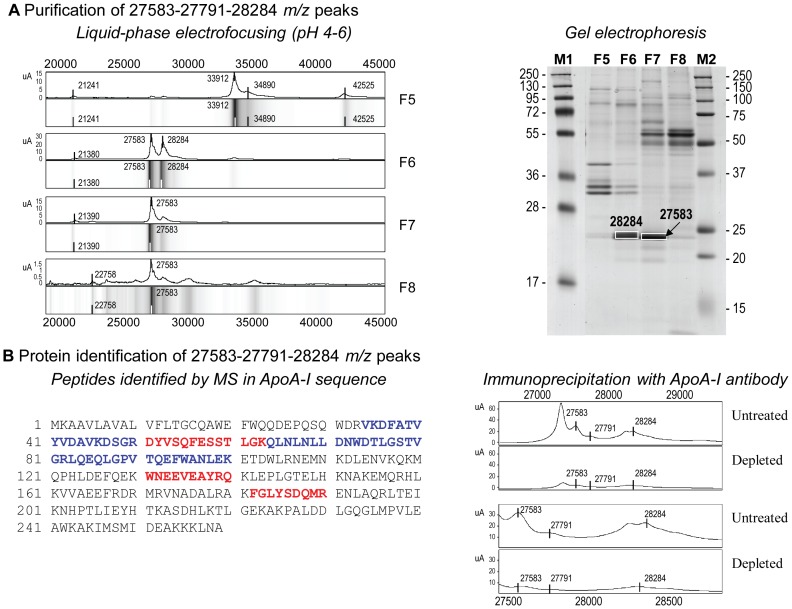
Purification and identification of the proteins corresponding to the 27583, 27791, and 28284 *m/z* peaks. (A) SELDI-TOF-MS protein spectrum of fractions F5, F6, F7, and F8 from MicroRotofor® cell, by a pH gradient 4–6 (left panel). Each fraction (F5, F6, F7, and F8) was analyzed on NU-PAGE 10% coomassie blue stained-gel. The bands corresponding to the 27583, 27791, and 28284 *m/z* peaks were framed (right panel). (B) Peptides identified by mass spectrometry corresponding to apolipoprotein A-I. Aminoacids indicated in red corresponds to peaks 27583 and 27791, and those indicated in blue correspond to peak 28284 *m/z* (left panel). SELDI-TOF-MS protein spectra of crude (untreated) and immunodepleted plasma with ApoA-I antibody (depleted) showed the decrease in 27583, 27791, and 28284 *m/z* peaks following immunodepletion, validating the identification (right panel).

Purification of the three peaks by liquid-phase IEF and gel electrophoresis (**Figure 2A**) combined with mass spectrometry successfully identified the peaks as being apolipoprotein A-I (ApoA-I) (**Figure 2B** **,** **Table 2**). We verified the identification of ApoA-I peaks using a specific polyclonal antibody. Immunodepletion significantly reduced the three peaks (**Figure 2B**), thereby confirming the identification of 27583, 27791, and 28284 *m/z* peaks as ApoA-I.

**Table 2:** Peak list of mass spectra corresponding to the SELDI peaks.

Peaks	Accession	Protein	Mass of peak	%	Probability
*m/z*	number	name	(Daltons)	coverage	score
27583	P04639	Apolipoprotein	1132.5186	11	47
27791		A-I	1195.5419		
			1460.7139		
28284	P04639	Apolipoprotein	1132.5176	32	360
		A-I	1195.5486		
			1227.6107		
			1454.7744		
			1460.6777		
			1879.8834		
			2129.1015		
			2130.0910		

**Figure 3:**
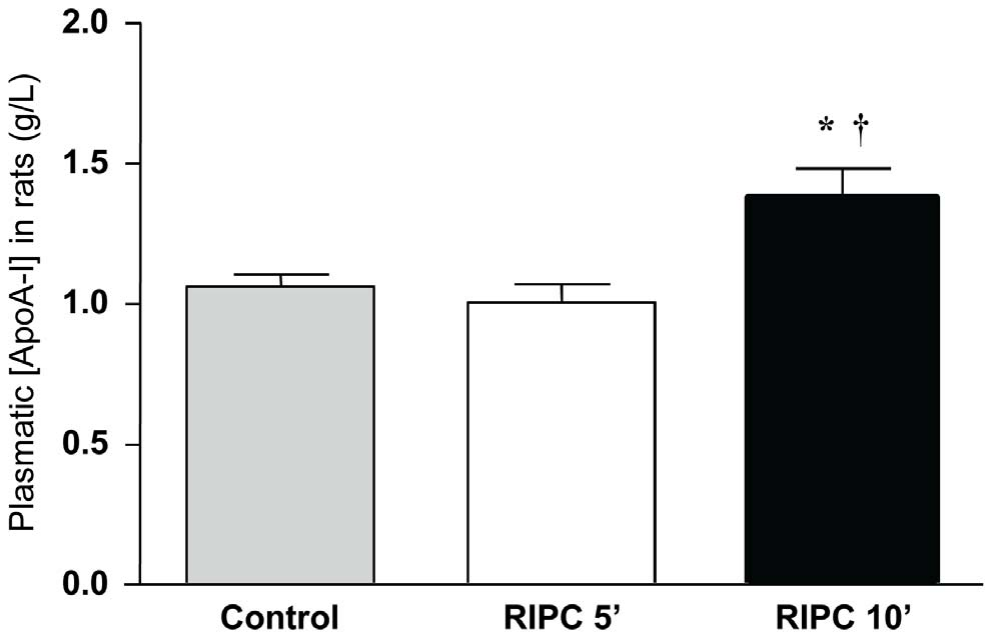
Validation of proteomic analysis by ELISA quantification of ApoA-I in rat plasmas. Results are expressed as mean ±SEM. *p<0.05 *vs.* RIPC 5′, †*p*<0.05 *vs.* Control, n = 10 in each group.

ELISA was used to verify the concentrations of plasma ApoA-I in the three groups of rats that were used for proteomic analysis. Plasmatic levels of ApoA-I were found to be upregulated in RIPC 10′ group (**Figure 3**), as detected via SELDI profiling. Undeniably, a 30.2% increase was observed between Control and RIPC 10′ groups (1.06±0.04 *vs.* 1.38±0.11 g/L, respectively; *p* = 0.01). In line with the SELDI-TOF-MS study, no significant release of ApoA-I was visualized in RIPC 5′ when compared to Control (1.07±0.09 *vs.* 1.07±0.04 g/L, respectively; *p* = 0.48).

### Cardioprotective Effect of ApoA-I as RIPC in MI Rats

**Figure 4:**
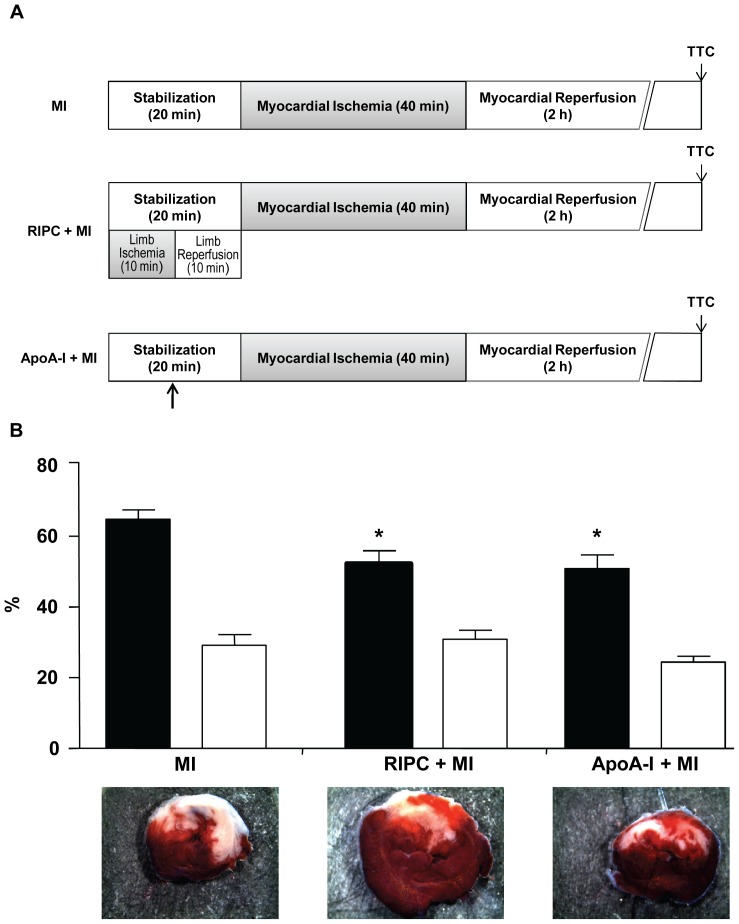
ApoA-I reduces infarcted area in rats submitted to ischaemia-reperfusion protocol. (A) Experimental protocol. All groups were subjected to 40-minute coronary occlusion followed by 2-hour reperfusion. RIPC was achieved by 10-minute limb ischemia followed by 10-minute reperfusion. ApoA-I (10 mg/kg) was administered 10 minutes before coronary artery occlusion (*arrow*). (B) Representative pictures of mid- ventricular 2,3,5-triphenyltetrazolium chloride-stained sections and bar graph showing infarct size expressed as a percentage of area at risk (black bars), and area at risk as a percentage of total left ventricular area (white bars). Results are given as mean ±SEM. *p<0.05 *vs.* MI group, n = 8–11 in each group.

In Study 2, rats were exposed to 40 minutes of myocardial ischemia followed by 2 hours of reperfusion. Three groups were constituted: Control (MI, myocardial I/R alone), RIPC+MI (10-min limb ischemia followed by 10-min reperfusion initiated 20 minutes prior to coronary artery occlusion), and ApoA-I+MI (ApoA-I injection 10 minutes prior to coronary artery occlusion), as illustrated in **Figure 4A**. The mortality rate was similar in all MI groups: 18% in MI, 15% in RIPC+MI, and 20% in ApoA-I+MI, with *p = *0.96. As shown in **Figure 4B**, the ischemic area induced by LAD ligation (AAR%LV) did not differ among the three groups (MI = 29.18±2.96%, n = 9; RIPC+MI = 30.90±2.54%, n = 11; ApoA-I+MI = 24.41±1.63%, n = 8; *p* = 0.21). RIPC induced a smaller infarct size when compared to untreated MI rats (INF%AAR = 52.21±3.72% in RIPC+MI *vs.* 64.86±2.57% in the MI alone group; *p*<0.05). Similarly, ApoA-I injection decreased infarct size (INF%AAR = 50.91±3.81% in ApoA-I+MI *vs.* 64.86±2.57% in MI alone; *p<*0.05), but infarct size did not significantly differ between the two treated groups (*p* = ns) (**Figure 4B and 4C**).

## Discussion

Since the initial studies that reported the capability of protecting the heart by applying ischemic preconditioning stimuli to an organ or tissue remote from the heart [2], the successful use of non-invasive transient upper-limb ischemia as a stimulus has greatly facilitated the translation of RIPC into a variety of clinical settings in acute I/R injury [7], . However, the mechanism through which RIPC exerts its protection against subsequently sustained cardiac I/R injury is still unclear. Involvement of three inter-related events is the widely accepted theory [6], [29], [30]. Firstly, brief sequences of ischemia to the remote tissue are believed to generate endogenous autacoids. Secondly, the protective signals need to be conveyed from the remote tissue to the myocardium. This step may involve several mechanisms, including blood-borne factors [13], [15], [16], [31], [32], neuronal pathways [3], [33], and systemic response [34]. Thirdly, the protective signals may activate several intracellular survival signaling pathways in the myocardium [29], [35]. A large body of evidence supports the concept of blood-borne factor(s) conveying the cardioprotective signal from the remote tissue to the heart. Indeed, a period of reperfusion to the remote preconditioned organ is required to insure protection after RIPC, suggesting that the reperfusion period may be needed to washout humoral factors generated by RIPC [3], [14], [36]. Moreover, whole blood taken from a rabbit that underwent ischemic preconditioning of both the heart and kidney was shown to be able to decrease infarct size when transfused into an untreated rabbit [13]. Similarly, coronary effluent from isolated rabbit hearts preconditioned by repetitive ischemia transferred protection to another acceptor isolated rabbit heart [15]. In another study, acutely transplanted *(i.e.,* denervated) hearts were preconditioned by remote limb stimulus in pigs, suggesting that a humoral factor was responsible for protection [16]. Several studies have reported the implication of endogenous factors in cardioprotective mechanisms, including bradykinin [37], adenosine [14], opioids [38], endocannabinoids [39], and erythropoietin [40]. However, to date, the actual identity of circulating humeral factors remains unknown.

In this study, the proteomic SELDI-TOF-MS approach detected a plasmatic up-regulation of ApoA-I in rats exposed to 10-min limb ischemia and 10-min reperfusion. In a previous study, Lang *et al*. already attempted to identify the humoral factors that convey the preconditioning signal from the remote organ to the heart using proteomic methods [41]. In the Lang *et al.* study, the RIPC stimulus was induced in rats through 10-min renal artery occlusion period followed by 20-min reperfusion. Blood samples were then analyzed using two-dimensional gel electrophoresis (2-DE), with 10 spots found to be differentially expressed in the control and RIPC groups. While using MALDI-TOF-MS, the authors identified only four protein spots, three of which corresponded to up-regulated albumin fragments, while one was identified as down-regulated liver regeneration-related protein in the preconditioned group. None of these proteins exhibit a known cytoprotective function. Several points may explain why ApoA-I was found not to be a potential RIPC factor in the Lang *et al.* study. Different RIPC models were used in both studies. We employed limb ischemia with femoral artery clamping, while Lang e*t al*. used renal artery clamping. Additionally, the timing of blood sampling differed: 10 minutes after limb reperfusion versus 20 minutes after renal reperfusion. One could speculate that the increased plasmatic ApoA-I levels display a Gauss curve with a maximum at 10 min, as we did not detect any increase at 5 min, whereas the Lang’s samples were analyzed after 20 minutes of reperfusion. In a recent paper, Hepponstall *et al.* examined changes in the plasma proteome of five healthy human volunteers exposed to four cycles of 5-min forearm ischemia followed by 5-min reperfusion [42]. Interestingly, ApoA-I was up-regulated at 15 minutes and down-regulated 24 hours following RIPC in comparison with baseline samples.

ApoA-I is the major protein component of high-density lipoproteins (HDL). The protective effect of HDL is mainly attributed to their capacity to dislodge lipids from atherosclerotic plaques [43]. However, HDL could also acutely protect the heart against I/R injury. Intravenous injection of human HDL, 30 minutes before transient coronary ligation, reduced infarct size by 20% in mice [44]. HDL suppressed leucocyte adhesion to the endothelium and protected cardiomyocytes against apoptosis. L-nitroarginine methyl ester (L-NAME) administration before I/R completely eliminated the protection afforded by HDL, underlining the major role played by nitric oxide (NO) in the cardioprotective mechanism. Another study demonstrated that single intravenous injection of synthetic HDL prior to myocardial ischemia increased NO production and suppressed reperfusion-induced arrhythmias through an Akt/ERK/NO pathway in endothelial cells [19]. Synthetic HDL was made of phophatidylcholine and ApoA-I. Similarly, Rossoni *et al.* showed improved functional post-ischemic recovery of isolated rat hearts through the administration of similar synthetic HDL before or after ischemia [45]. Beneficial effects were attributed to the scavenging of myocardial released TNF-alpha. Furthermore, ApoA-I alone, administered intravenously 30 minutes before occlusion of the renal pedicles, decreased renal I/R injury by inhibiting inflammatory cytokine release (TNF-alpha, IL-1beta) as well as neutrophil infiltration and activation [20]. In addition, Gu *et al.* reported that administration of ApoA-I before the onset of reperfusion of myocardial infarction decreased creatine kinase release, diminished the production of TNF-alpha and IL-6 in cardiac tissue, and suppressed the endothelial expression of ICAM-1 in addition to neutrophil adherence and migration [18].

This study provided further evidence that the heart can be preconditioned by single ApoA-I administration, recapitulating RIPC-induced cardioprotection. It is worthy to note that the recently reported mechanisms using HDL or ApoA-I injection in I/R models were also described in RIPC. Accordingly, RIPC was shown to be associated with NO release [46] and anti-inflammatory effects [47].

Finally, a previous study has shown that the humoral factors, induced by limb RIPC, are likely to be hydrophobic with a molecular mass <15 kDa [31]. A recent study reported that the coronary effluent from ischemic preconditioned hearts contained hydrophobic protective factors with a molecular mass below or close to 30 kDa, with at least one of them having a molecular mass >10 kDa [32]. The molecular mass of ApoA-I (28 kDa) [48] fits with these latest findings. Indeed, ApoA-I contains hydrophobic domains. The hydrophobicity of the C-terminal lipid-binding domain of ApoA-I is presumed to play a major role in promoting cholesterol efflux [49].

### Limits

The mechanism by which ApoA-I concentrations increase in the blood as early on as 10 minutes after limb reperfusion is still unknown, requiring further investigation. One could speculate that plasma ApoA-I level increase is not related to a new synthesis, but due to mobilization of existing ApoA-I in response to the RIPC stimulus. Our results were obtained in a rat I/R model using a single protocol of 10-min limb ischemia followed by 10-min reperfusion. This protocol was previously validated in rats by our group along with other groups [35], [50]. However, our results need to be confirmed using other RIPC stimuli, and further studies are necessary in order to ascertain whether ApoA-I is involved in RIPC-induced cardioprotection in other animal species as well as in humans with comorbidities, such as diabetes or lipid disorders.

## Conclusion

Our findings suggest that ApoA-I may be a protective blood-borne factor involved in the RIPC mechanism. RIPC was associated with a plasmatic increase in ApoA-I, and ApoA-I injection prior to myocardial I/R recapitulated cardioprotection offered by RIPC. Further studies are needed to confirm this data in other experimental models and in clinical settings.
